# Pathological Yawning in Patients with Acute Middle Cerebral Artery Infarction: Prognostic Significance and Association with the Infarct Location

**DOI:** 10.4274/balkanmedj.galenos.2019.2019.7.49

**Published:** 2019-12-20

**Authors:** Aslı Aksoy Gündoğdu, Atilla Özcan Özdemir, Serhat Özkan

**Affiliations:** 1 Department of Neurology, Tekirdağ Namık Kemal University School of Medicine, Tekirdağ, Turkey; 2 Department of Neurology, Eskişehir Osmangazi University School of Medicine, Eskişehir, Turkey

**Keywords:** Anterior cerebral circulation, infarct location, middle cerebral artery infarction, pathological yawning, prognosis

## Abstract

**Background:**

Pathological yawning is a compulsive, frequent, repetitive yawning triggered by a specific reason besides fatigue or boredom. It may be related to iatrogenic, neurologic, psychiatric, gastrointestinal, or metabolic disorders. Moreover, it could also be seen in the course of an ischemic stroke.

**Aims:**

To determine whether pathological yawning is a prognostic marker of middle cerebral artery stroke and evaluate its relationship with the infarct location.

**Study Design:**

Cross-sectional study.

**Methods:**

We examined 161 patients with acute middle cerebral artery stroke, consecutively admitted to emergency department. Demographic information, stroke risk factors, stroke type according to Trial of Org 10172 in Acute Stroke Treatment classification, blood oxygen saturation, body temperature, blood pressure, heart rate, glucose levels, daytime of stroke onset, National Institutes of Health Stroke Scale score (National Institutes of Health Stroke Scale score, at admission and 24 h), modified Rankin scale (at 3 months), and infarct locations were documented. Pathological yawning was defined as ≥3 yawns/15 min. All patients were observed for 6 hours to detect pathological yawning. National Institutes of Health Stroke Scale score >10 was determined as severe stroke. The correlation between the presence of pathological yawning and stroke severity, infarct location, and the short- and long-term outcomes of the patients were evaluated.

**Results:**

Sixty-nine (42.9%) patients had pathological yawning and 112 (69.6%) had cortical infarcts. Insular and opercular infarcts were detected in 65 (40.4%) and 54 (33.5%) patients, respectively. Pathological yawning was more frequently observed in patients with cortical, insular, and opercular infarcts (p<0.05). Pathological yawning was related to higher National Institutes of Health Stroke Scale scores. Patients with severe stroke (National Institutes of Health Stroke Scale score ≥10) presented with more pathological yawning than those with mild to moderate strokes (p<0.05). The clinical outcomes and mortality rates showed no significant relationship with the occurrence of pathological yawning.

**Conclusion:**

Pathological yawning in middle cerebral artery stroke was associated with stroke severity, presence of cortical involvement, and insular and opercular infarcts. However, no association was found with long-term outcome and mortality.

Stroke is a common neurological disease, which is the major cause of disability and mortality in both genders, and has an accelerating frequency due to an increase in life expectancy in adult age group (1,2). A variety of factors influence the outcome of stroke, including age, gender, stroke severity, early rehabilitation, stroke etiology, infarct location, rehabilitation, cognitive decline, aphasia, depression, and comorbid diseases (3). Being able to predict the prognosis of stroke makes the length of hospital stay or long-term costs manageable and may reduce the economic burden of stroke. Studies providing and comparing prognosis, survival, and recurrence data on stroke allows clinicians to identify high-risk patients for stroke recurrence and stroke-related death, researchers to plan clinical trials to develop new strategies, and public health policy-makers to bear a clearer picture of the social impact of ischemic stroke.

Yawning is a very common stereotyped motor behavior physiologically observed in humans, other mammals, and numerous animal species (4,5). Healthy humans may yawn 0-28 times/day, and this frequency of physiological yawning may vary according to age, circadian rhythm, arousal, decreased attention, boredom, fatigue, hunger, satiety, and before- and after-sleep episodes (6,7). Former studies revealed that paraventricular nucleus of the hypothalamus, hippocampus, reticular activating system in the brainstem, cervical spinal cord (phrenic nerve C1-4), intercostal muscles, oxytocin, acetylcholine, dopamine, glutamate, serotonin, gamma-aminobutyric acid, adrenergics, adrenocorticotropic hormone, and α-melanocyte-stimulating hormone are involved in the occurrence and mediation of yawning (6,7,8).

Cortical involvement of yawning has been defined by recent studies but not been fully demonstrated yet (5,7). Frequent, repetitive, and compulsive yawning episodes are termed as excessive, abnormal, or pathological. Besides the physiological factors such as fatigue, boredom, or contagion; pathological yawning (PY) is found to be triggered by various iatrogenic causes and several metabolic, gastrointestinal, psychiatric, or neurological diseases (9,10,11,12). PY has been reported in numerous neurological conditions including parkinsonism, Parkinson’s disease, progressive supranuclear palsy, Huntington disease, myasthenia gravis, bulbar amyotrophic lateral sclerosis, multiple sclerosis, neuromyelitis optica spectrum disorders, migraine aura, vasovagal syncope, narcolepsy, brain tumor, encephalitis, intracranial hypertension, stroke, Chiari malformation type I, epilepsy, stress, and anxiety disorders (6,9,10,11,12,13,14,15,16,17,18,19,20,21,22,23,24,25). Although PY in brainstem and anterior circulation ischemic stroke has been previously reported in the literature, to date, the exact mechanism of cortical network remains to be established by functional neuroimaging studies. Some recent studies concluded that ischemic lesions of the posterior insula and caudate nucleus induce PY. Yet, there is no enough clinical data in humans regarding PY in anterior circulation stroke and the frequency or prognostic effect of PY on long-term prognosis and mortality rates of middle cerebral artery (MCA) strokes.

We hypothesized that PY may be a practical and easily detectable predictive phenomenon of outcome after acute ischemic stroke. In this study, we aimed to determine whether PY is a prognostic marker of MCA stroke and evaluate its relationship with the patient demographics, clinical parameters, infarct location, and outcome of the patients.

## MATERIALS AND METHODS

In this observational, cross-sectional study, we assessed 161 acute MCA stroke patients in a one-year period, who were consecutively admitted to emergency department and referred to our Neurology Stroke Unit within 24 hours of stroke onset. All patients were over 18 years of age, and only acute ischemic stroke patients were included in the study. Exclusion criteria included seizures, hypoglycemia, hypoxia, fever (>38 °C), usage of anesthetic agents, and an acute posterior circulation stroke or a history of it.

Demographic information, stroke risk factors, stroke type, daytime of stroke, blood oxygen saturation, body temperature, blood pressure, heart rate, glucose levels, receiving status of intravenous thrombolytic therapy, neurological and functional outcomes, and mortality status of the patients were documented. Stroke risk factors including hypertension, hyperlipidemia, atrial fibrillation, patent foramen ovale, diabetes mellitus, angina pectoris, coronary artery disease, peripheral artery disease, metabolic syndrome, and tobacco use were registered. A previous history of stroke or transient ischemic attacks was recorded. The usage of statins, antihypertensive (angiotensin-converting enzyme inhibitors, angiotensin II receptor blockers, beta blockers, and calcium-channel blockers), anticoagulant, antiplatelet, antiparkinsonian, and antidepressant drugs, and receiving status of intravenous thrombolytic therapy were noted. Routine hematological and biochemical peripheral blood analyzes were obtained on admission.

Daytime of stroke attacks between 00:00 and 06:00 hours were recorded as “increased sleepiness”. Clinical severity at baseline and 24 hours after symptom onset were assessed prospectively by using the National Institutes of Health Stroke Scale (NIHSS). Patients with NIHSS score ≥10 were considered to have severe neurological deficits. The modified Rankin scale was used to assess neurological and functional outcomes at 24 hours and 3 months after stroke. Prognosis was stratified according to the modified Rankin scale at 3 months: very favorable outcome as score 0-1, favorable outcome as score 0-2, and unfavorable outcome as score 3-6.

Cranial computed tomography and magnetic resonance imaging were performed in all patients. Hemispheric side, cortical, subcortical, frontal, temporal, parietal, insular, and opercular infarcts were recorded.

All patients underwent carotid Doppler ultrasonography, 12 lead electrocardiography, transthoracic echocardiography, transesophageal echocardiography, 24 hour Holter monitoring, computed tomography/magnetic resonance angiography, and digital subtraction angiography whenever indicated. Stroke type was determined using the Trial of Org 10172 in Acute Stroke Treatment criteria (26).

All patients were observed by neurologists for 6 hours after admission for the presence of PY. According to the criteria for abnormal yawning proposed by Singer et al. (24), count of yawns >3/15 minutes was determined as the cutoff number for PY. All findings of the patients were compared between two groups, the groups of patients with PY and of those without PY.

This study was approved by the Eskişehir Osmangazi University Hospital Ethics Committee (2012/159) and written informed consent was obtained from each patient.

### Statistical analysis

Statistical analysis of our study was performed by using SPSS program (Statistical package for social sciences, 24.0 windows; SPSS Inc., Chicago, IL, USA). A post-hoc power analysis of this study was performed on GPower program. Considering an alpha error of 0.05, a medium effect size (0.3), and the power of our study as 85%, a total sample size of 160 patients was found to be sufficient. Student’s t-test was used for the comparison of two independent groups with normally distributed data and Mann-Whitney U test was used for the abnormally distributed data. Categorical data were presented as frequency of occurrence and were analyzed by Pearson’s chi-square and two ratio tests. Continuous data were presented as mean ± standard deviation. A value of p<0.05 was accepted as the level of significance.

## RESULTS

Among the 161 patients included in our study, 81 patients were male (51.3%) and 80 female (49.7%). The mean age of the patients was 67.3±10.9 years (range: 18-85 years) and 69 (42.9%) of the 161 patients revealed PY. Table 1 gives the demographic and clinical characteristics of the patients. No significant relationship was found regarding age, sex, increased sleepiness, glucose levels, leukocyte counts (p=0.722, 0.82, 0.516, 0.715, and 0.401, respectively) and vascular risk factors (hypertension, diabetes mellitus, hyperlipidemia, coronary artery disease, congestive heart failure, valvular heart disease, atrial fibrillation, metabolic syndrome, tobacco use, and history of previous stroke) except for the history of previous transient ischemic attacks, between the patients with PY and those without PY (p=0.299, 0.504, 0.184, 0.265, 0.665, 0.857, 0.844, 0.061, 0.890, and 0.908, respectively).

The usage of statins, antihypertensive (angiotensin-converting enzyme inhibitors, angiotensin II receptor blockers, beta blockers, and calcium-channel blockers), anticoagulant, antiplatelet, antiparkinsonian, and antidepressant drugs, and the receiving status of intravenous thrombolytic therapy were recorded. Only one patient was using dopamine agonist medication for Parkinson’s disease. However, no PY was observed in this patient. Excluding the antiparkinsonian drugs, there was no significant relationship found between the usage of these drugs and PY (p=0.648, 0.776, 0.147, 0.111, 0.773, 0.738, 0.427, and 0.642, respectively).

Patients with PY presented with a higher NIHSS score compared with the patients without PY (p=0.019). Patients with severe stroke (NIHSS score ≥10) presented with more PY than those with mild to moderate stroke (NIHSS score <10) (p=0.004). PY was related to higher baseline NIHSS scores representing clinically severe patients (NIHSS score ≥10). A total of 32 (19.8%) patients died during follow-up period. The clinical outcomes (very favorable, favorable, and unfavorable) and mortality rates of the patients showed no significant relationship with the occurrence of PY. Clinical severity and outcomes of the patients are summarized in Table 2.

Hemispheric side of the infarcts or subcortical infarcts revealed no significant relationship with PY (p=0.237 and 0.772). However, PY was observed more frequently in patients with cortical, insular, and opercular infarcts (p=0.015, 0.046, and 0.008, respectively). Table 3 gives the neuroimaging findings (cerebral magnetic resonance imaging and computed tomography) of the patients.

## DISCUSSION

This observational study investigated whether PY affected the clinical outcome and mortality in patients with acute MCA stroke. We hypothesized that certain infarct locations in the anterior circulation system may facilitate PY, and the presence of PY may be considered as a prognostic factor for MCA strokes. Among our cohort of 161 patients, PY was observed in 69 (42.9%) patients and was likely to occur in patients with higher NIHSS scores. An equal distribution of gender was a strong aspect of our study. We found PY to be related with cortical involvement and insular and opercular infarcts. Our study revealed that PY is a common phenomenon among patients with MCA stroke and seems to be associated with stroke severity. However, no relationship was found regarding its effect on long-term outcome or mortality rates of the patients.

Evidences from former case reports and studies suggest that PY occurs frequently in the course of many neurological diseases (6,9,10,11,12,13,14,15,16,17,18,19,20,21,22,23,24,25). A limited number of studies have been reported on PY in acute ischemic stroke (9,23,24). Bauer et al. (27) stated that the patients with locked-in syndrome can elicit yawning movements involuntarily, despite a total paralysis of the volunteer bulbar muscles (27). Cattaneo et al. (9) published a case report of two patients with brainstem stroke who were presented with PY.

To date, only two studies have provided data concerning PY in anterior circulation stroke. The pilot study of Singer et al. (24) revealed that PY can be a sign of anterior circulation lesions. They observed PY in seven patients with anterior circulation strokes in MCA territory and hypothesized that PY occurs due to supratentorial lesions releasing the hypothalamic paraventricular nucleus from neocortical control mechanisms and increasing the activity of hippocampus and periamygdalar regions (24). A more recent study by Krestel et al. (25) investigated PY in 10 patients with acute anterior circulation stroke. Infarct regions and volumes were evaluated using magnetic resonance imaging lesion maps and diffusion weighted and apparent diffusion coefficient images. Intensity of the infarcts was found to be correlated with the period of abnormal yawning. They proposed that insular and caudate nucleus infarcts are responsible for PY (25).

The use of dopaminergic D2 agonists, imipramine, and selective serotonin reuptake inhibitors; morphine withdrawal; valproate overdose; and estrogen substitution may induce PY. Anesthetic agents lead to drowsiness and loss of consciousness (28). Yet, none of our patients were using these agents. Intravenous thrombolytic therapy has a positive impact on prognosis. However, we found no significant relationship between the patients who received thrombolytic therapy and the occurrence of PY.

It has been noted that PY is primarily triggered by low vigilance. However, PY can be seen even when there is no change in level of consciousness during stroke attacks. This may be because of the increased intracranial pressure secondary to stroke or the damage of particular cortico-subcortical circuits and the disruption of the connections between the reticular formation that regulate alertness in the brain stem. As the clinical severity of stroke increases, PY is observed more frequently (5,6,7). Krestel et al. (25) found a significant correlation between the period of PY and stroke severity (25). Factors such as low vigilance, increased brain temperature, intracranial hypertension, deterioration of homeostasis, and damage of more neuroanatomical structures including cortico-subcortical circuits may be the possible causes of PY (5,6,7).

This study has several limitations. First, during observation period, we could not video-record the patients. Thus, the duration or the distinctive features of yawning attacks could not be measured quantitatively. Moreover, despite the cutoff yawning count for PY (≥ 3yawns/15 min) was determined after two previous studies (24,25), physiological yawning may also occur at the same frequency. And finally, sleepiness scale tests could not be performed in the aphasic or clinically serious patients. This situation led us insufficient data regarding increased sleepiness or drowsiness in the patients.

Further studies measuring the neurotransmitter and neurohormone levels released during PY attacks in acute stroke or those using improved neuroradiological tools such as tractography are required to discover the exact pathophysiological mechanism and neural pathways responsible for PY. The causative factors that trigger PY in acute stroke involving cortical brain areas and clinical significance of PY still remain to be clarified.

To the best of our knowledge, the present study is the first one analyzing the clinical and radiologic findings of PY in acute MCA stroke with larger human cohort, including findings regarding long-term outcomes and mortality rates of the patients with PY. Consistent with the existing evidence, our study revealed that cortical involvement, and opercular and insular infarcts trigger PY. Supporting statistically, we established the clinical significance of PY and could evaluate its prognostic role in MCA stroke. Notwithstanding its connection with the clinical severity, PY reveals no significant predictive value for clinical outcome of patients with MCA stroke.

## Figures and Tables

**Table 1:**
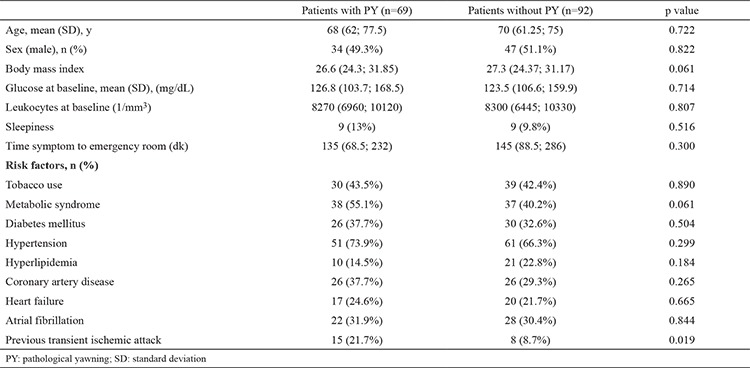
Demographics and clinical characteristics of the patients

**Table 2:**
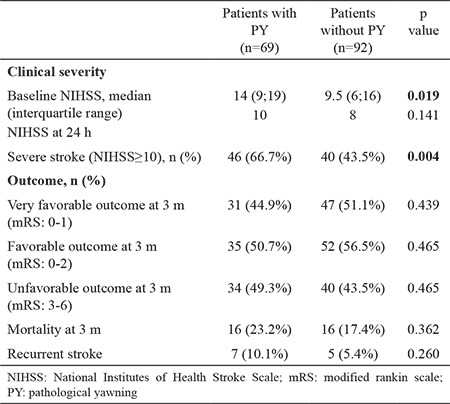
Clinical severity and outcome of the patients

**Table 3:**
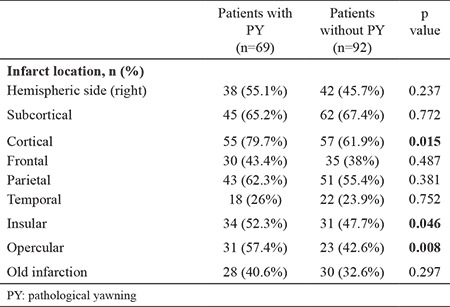
Neuroimaging findings of the patients
